# Genomic analysis of the class *Phycisphaerae* reveals a versatile group of complex carbon-degrading bacteria

**DOI:** 10.1007/s10482-024-02002-7

**Published:** 2024-07-23

**Authors:** Wouter B. Lenferink, Theo A. van Alen, Mike S. M. Jetten, Huub J. M. Op den Camp, Maartje A. H. J. van Kessel, Sebastian Lücker

**Affiliations:** Department of Microbiology, Radboud Institute for Biological and Environmental Sciences, Radboud University, Heyendaalseweg 135, 6525 AJ Nijmegen, The Netherlands

**Keywords:** Carbon metabolism, CAZYmes, Phycisphaerae, Planctomycetes

## Abstract

Bacteria of the phylum *Planctomycetota* have received much attention over the years due to their unique cell biology and potential for biotechnological application. Within the phylum, bacteria of the class *Phycisphaerae* have been found in a multitude of environmental datasets. However, only a few species have been brought into culture so far and even enrichments are scarce. Therefore, very little is known about their lifestyle, which has hindered efforts to estimate their environmental relevance. Here, we analysed all medium- and high-quality *Phycisphaerae* genomes represented in the genome taxonomy database to learn more about their physiology. We combined automatic and manual annotation efforts to provide a bird’s eye view of their diverse energy metabolisms. Contrasting previous reports, we did not find indications for the presence of genes for anaerobic ammonium oxidation in any *Phycisphaerae* genome. Instead, we found that many members of this class are adapted to a facultative anaerobic or strictly fermentative lifestyle and may be specialized in the breakdown of carbon compounds produced by other organisms. Based on these findings, we provide a practical overview of organic carbon substrates predicted to be utilized by *Phycisphaerae* families.

**Supplementary Information:**

The online version contains supplementary material available at 10.1007/s10482-024-02002-7.

## Introduction

The *Planctomycetota* are a large phylum of bacteria that exhibit some remarkable cellular features and are important in both carbon and nitrogen cycling. Perhaps most famous within this phylum are members of the class *Ca.* Brocadiia, which have been exclusively found to perform anaerobic ammonium oxidation (anammox). This process is considered fundamental for the loss of nitrogen from marine ecosystems and has found application in the wastewater treatment industry (Kartal et al. 2010; Kuypers et al. 2018; Lam & Kuypers 2011). The *Ca.* Brocadiia share a unique organelle-like structure called the anammoxosome (van Niftrik et al. 2008) required for the anammox process to function. Another relatively well-studied class within the *Planctomycetota* is the *Planctomycetia*. Members of this class are often found in close association with macroalgae and sponges and are generally found in marine and brackish environments (Wiegand et al. 2019). A high number of carbohydrate-active enzymes (CAZymes) and their ability to grow on complex carbon substrates suggest that *Planctomycetia* are important to carbon cycling in aquatic environments. Furthermore, *Planctomycetia* genomes often contain several biosynthetic gene clusters (BGCs), necessary to produce bioactive small molecules (Kallscheuer and Jogler 2021) that have functions in pigmentation but may also be an untapped source of therapeutics.

The lesser-known class of *Phycisphaerae* is found in a much larger variety of habitats than the *Ca.* Brocadiia and *Planctomycetia*. *Phycisphaerae* have been found in metagenomic datasets in marine and freshwater systems, sediments, soils, associated with macroalgae, coal bed mines, and multiple engineered ecosystems including wastewater treatment and solid-state fermentation reactors (e.g., Dedysh et al. 2020; Robbins et al. 2016; Spring et al. 2018; Stultiens et al. 2020). However, despite their ubiquitous presence, only 11 species are currently described, and most data originates from metagenomic studies on environmental samples. This is in stark contrast to the over 100 described species of *Planctomycetia* and the highly enriched cultures available for several anammox species. The lack of cultured representatives makes it difficult to study the metabolic processes that *Phycisphaerae* may carry out. Moreover, the ecological role of different *Phycisphaerae* species in natural environments remains elusive. Consequently, their presence often leads to speculation in literature with little to no follow-up. For example, *Phycisphaerae* have been implicated with important roles in carbon cycling (Wang et al. 2015) or processes such as sulphate- and iron-dependent anaerobic ammonium oxidation (Suarez et al. 2023). Still, there is a general lack of experimental evidence to support such hypotheses.

Ideally, hypotheses about the lifestyle of *Phycisphaerae* bacteria should be tested in pure or highly enriched cultures. However, the *Planctomycetota* have proven to be difficult to culture due to several challenges. First, we know little about the substrates and culture conditions that they prefer. Secondly, *Planctomycetota* have infamously long generation times, sometimes taking weeks to duplicate. In the case of *Planctomycetia*, where N-acetylglucosamine was discovered as a selective substrate, getting pure cultures can still take months (Wiegand et al. 2019). Equally, little is known about the metabolism of *Phycisphaerae*, making it difficult to develop cultivation strategies for this group. On the other hand, *Phycisphaerae* are well represented in many environmental metagenomic datasets. In the Genome Taxonomy Database (GTDB) alone, *Phycisphaerae* are represented by 881 genomes (release 202; Chaumeil et al. 2020). Therefore, we set out to combine the available genomic information of *Phycisphaerae* with reports of isolates in the literature. With this approach, we aim to attribute metabolic lifestyles to the different families represented in this class. In addition, we aim to provide the field with practical considerations to assist in the isolation of *Phycisphaerae* bacteria.

## Materials and methods

### Genomes included in this study

We gathered all accession numbers classified as “c__Phycisphaerae” from the GTDB database (version R202) and downloaded the latest assemblies from the NCBI FTP server. Metadata from these genomes was obtained from Biosample using NCBI Entrez in R (“rentrez” package). The environmental classification as oxic or anoxic was inferred from this metadata. Environments where limited amounts of oxygen were supplied (e.g., in chemostat enrichments) were classified as oxic. Additionally, we included genomes from in-house metagenomic datasets obtained from a bioelectrochemical system (indicated as AOM_BES5 and described in Ouboter et al. 2022), an enrichment of Fe-dependent anaerobic methane oxidizers (AOM_Fe1; Ettwig et al. 2016), a brewery wastewater treatment plant (DAMOX_Bav1; Stultiens et al. 2020), a recirculating aquaculture system biofilter (COM_RAS1, COM_RAS2; Van Kessel et al. 2015), a coupled two-reactor set-up for the enrichment of ammonia- and nitrite-oxidizers (TNR_A1, TNR_A2, TNR_N1, TNR_N2, TNR_N3, TNR_N4; Sakoula et al. 2022), the wall biofilm of a drinking water treatment plant rapid sand filter (COM_TrWB2; Poghosyan et al. 2020), and an anammox enrichment (AMOX_SL1; Schmid et al. 2003).

All GTDB- and in-house-derived genomes were quality filtered based on CheckM2 (Chklovski et al. 2022) scores (≥ 70% completeness, ≤ 10% contamination) and ≤ 500 contigs, and dereplicated using dRep version 2.4.2 (Olm et al. 2017). An additional 6 genomes were removed because they either contained 16S rRNA sequences with non-*Phycisphaerae* BLAST hits or were classified by GTDB-Tk (see below) outside this class. In total, this yielded 187 genomes which were analysed further.

### Phylogeny and taxonomic assignment

All in-house genomes were taxonomically assigned using GTDB-Tk (Chaumeil et al. 2020). For phylogenetic inference, alignments were made based on 81 bacterial core genes from all genomes included in this study, using the Up-to-date Bacterial Core Gene 2 (UBCG2) pipeline (Kim et al. 2021b). The final phylogenetic trees were compiled from the UBCG2 core gene alignment using IQTree 1.6.12 (Nguyen et al. 2015) using ModelFinder (Kalyaanamoorthy et al. 2017) to infer the best evolutionary model. Three genomes of the *Verrucomicrobiaceae* were included to root the tree (*Roseimicrobium gellanilyticum*, *Verrucomicrobium spinosum*, and *Prosthecobacter fusiformis*). Visualization of trees and annotations were done with the ‘treeio’ (Wang et al. 2020) and ‘ggtree’ package (Yu 2020) in RStudio. The taxonomic assignment of JAAYCJ01 and FEN-1346 as orders was additionally examined using EzAAI 1.1 (Kim et al. 2021a), which provides pairwise average amino acid identity (AAI) values.

### Genome annotation

Gene calling and annotation of all genomes were performed using the Anvi’o 7 metagenomics pipeline (Eren et al. 2015). In Anvi’o, gene calling was done with Prodigal 2.6.3 (Hyatt et al. 2010), followed by annotation using the reference sets for single-copy marker genes, rRNA, and tRNA, as well as the NCBI COG20 database in sensitive mode (Galperin et al. 2021), and the KEGG KOfam database (Aramaki et al. 2020). Completeness of KEGG pathways was estimated using ‘anvi-estimate-metabolism’ at default settings. Annotation of carbohydrate-active enzymes (CAZymes) and CAZyme gene clusters (CGCs; genomically linked clusters of CAZyme genes) were performed using dbCAN2 and CGCFinder (Zhang et al. 2018). Substrate prediction of CAZymes in CGCs was performed using dbCAN4 (Zheng et al. 2023). CAZyme predictions were considered true positives when predictions were congruent between at least 2 tools included in dbCAN, i.e., dbCAN sub, HMMer, or DIAMOND. The function predictions were further filtered to keep only those CAZYme annotations that were part of a CGC. Semi-automatic annotation of nitrogen, sulphur, and iron cycling genes was performed using HMM profiles included in Metascan (Cremers et al. 2022). To investigate the use of alternative electron acceptors, we used HMMs for nitrate reductase (*narGHI*), nitrite reductase (*nirK* and *nirS*), nitric oxide reductase (*norBC*), and nitrous oxide reductase (*nosZ*), as well as for sulphate (*aprAB* and *sat*) and sulphite reductases (*asrABC* and *dsrAB*). We also included HMMs outer membrane *c*-type cytochromes (*mtrBC*), genes involved in (anaerobic) ammonia/ammonium oxidation (*amoA*, *hao, hzsABC*, *hdh*), and urea utilization (*ureABC* and fused *ureAB*). For consistency, we regarded HMM hits to be reliable with a bitscore ≥ 40 and an E-value ≤ 1e^−15^. The bitscore cutoff was based on recent findings in *Phycisphaera* by (Suarez et al. 2023) for the discovery of divergent sequences. Further manual annotation of genes not included in KEGG modules was performed using BLASTp (Camacho et al. 2009) or HMMER 3.1b2 (http://hmmer.org) when HMMs were available from PFAM (Mistry et al. 2021).

## Results

**Fig. 1:**
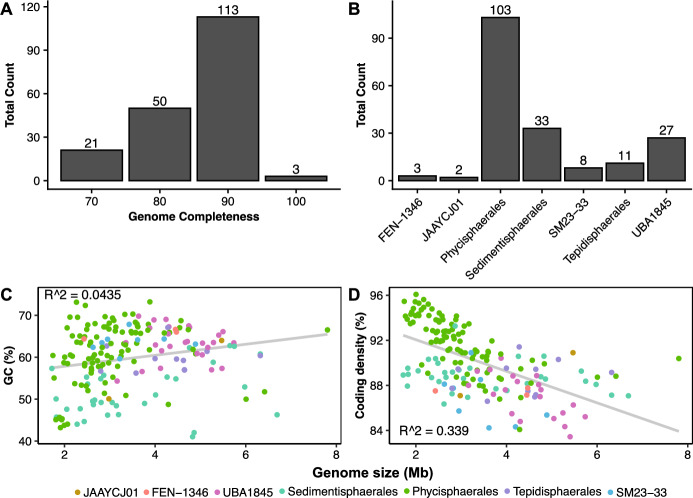
Genome statistics for the 187 *Phycisphaerae* genomes analysed. **A** Genome completeness counts rounded to 10%. **B** Number of genomes included per GTDB-Tk-assigned order. **C** GC content (%) as a function of genome size in million base pairs (Mb). **D** Coding density (%) as a function of genome size. Colours represent GTDB-Tk-assigned orders

**Fig. 2:**
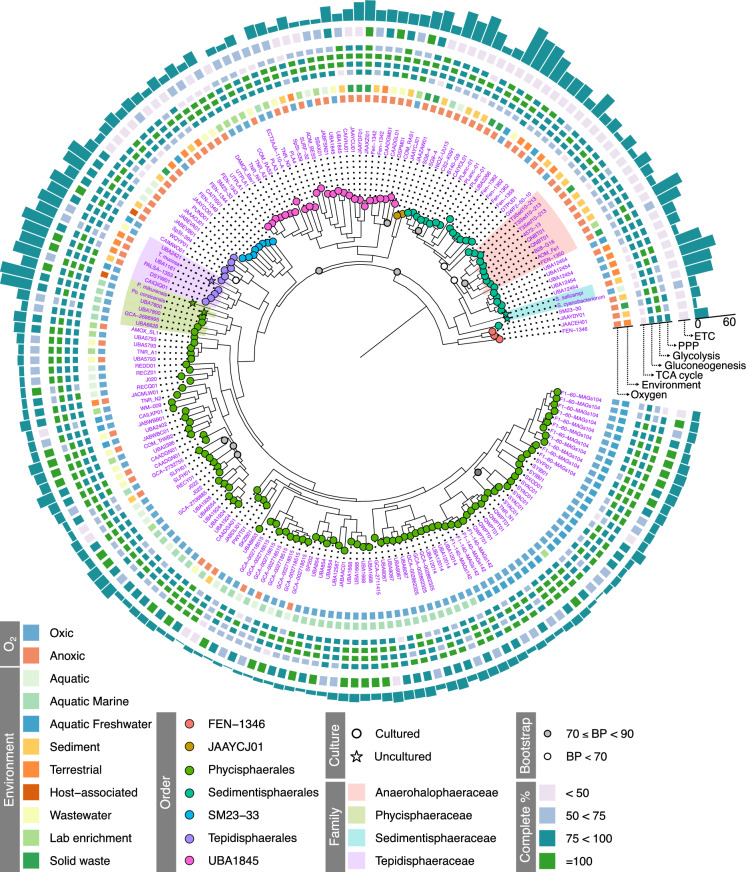
Phylogenetic tree of the *Phycisphaerae* decorated with metadata from NCBI BioSample, genome annotation, and taxonomy by GTDB-Tk. The tree was rooted with 3 representatives of the *Verrucomicrobiaceae*. Bootstrap percentages (BP) ≥ 90% are not shown. Inner tiled ring: environment and oxygen availability of the sample origin. Middle tiled ring: completeness of central metabolic pathways based on KEGG annotation. ETC, electron transport chain complex I, III, IV, and V; TCA cycle, tricarboxylic acid cycle; PPP, pentose phosphate pathway. Outer tiled ring: completeness of the electron transport chain (ETC) based on KEGG and manual annotation. Bar chart outer ring: number of unique carbohydrate-active enzyme gene clusters present in the genome

The quality screening and dereplication of all *Phycisphaerae* genomes available in-house and in the GTDB R202 database yielded a total of 187 genomes that were retained for further analysis (Table S1). 113 of these were of an estimated completeness ≥ 90% (Fig. 1). Genome sizes ranged from 1.71 to 7.8 Mb, coding densities from 83 to 96%, and GC content from 41 to 73%. Representatives of the orders *Phycisphaerales*, *Sedimentisphaerales*, UBA1845, *Tepidisphaerales*, SM23-33, FEN-1346, and JAAYCJ01 were present in the dataset (in order of genome count in our dataset), including genomes of the isolates *Phycisphaera mikurensis*, *Poriferasphaera corsica*, *Tepidisphaera mucosa, Sedimentisphaera salicampi,* and *Sedimentisphaera cyanobacteriorum*. In general, the phylogenetic placement of all *Phycisphaerae* orders was well-supported (Bootstrap ≥ 90%). Low support (Bootstrap < 70%) was found only for families within the *Sedimentisphaerales*. Our genome set originated from a broad range of habitats, including aquatic, terrestrial, and engineered environments (Fig. 2) and included more extreme environments such as hypersaline lakes, hydrothermal vents, and permafrost habitats. Furthermore, the dataset comprised both oxic and anoxic environments.

### *Phycisphaerales*

The *Phycisphaerales* contained most sequenced genomes in our dataset and were represented by 103 genomes of mainly oxic freshwater and marine habitats. The order was subdivided into 2 families, namely the *Phycisphaercaceae* (6 genomes) and SM1A02 (97 genomes). The *Phycisphaeraceae* contained genomes with the largest genome size (5.29 ± 1.72 Mb), which was only 2.84 ± 0.64 Mb for SM1A02. SM1A02-affiliated genomes had an average coding density of 92 ± 2%, the highest of all *Phycisphaerae*. Most *Phycisphaerales* genomes contained a near-complete electron transport chain (ETC). In most cases the NADH dehydrogenase was predicted to be missing due to the absence of *nuoF* and *nuoG* annotations. No *Phycisphaerales* genome contained the cytochrome *bc*_1_ complex; instead, they encoded the alternative complex III (ACT). Surprisingly, except for *Po. corsica*, all *Phycisphaeracaea* genomes lacked the cytochrome *bd*-type quinol oxidase, or *caa*_3_- and *cbb*_3_-type cytochrome *c* oxidases. Alternative electron acceptors for *Phycisphaerales* could be nitrate and nitrite, as several genomes encoded *narGH*, *nirK*, and *norB* (Figure S2). Furthermore, some *Phycisphaeraceae* genomes contained an octaheme cytochrome *c* protein related to octaheme nitrite reductase (Onr; Ferousi et al. 2021), which was detected with the Hao HMM. However, they lack *nosZ* genes. The presence of the *aprA* and *sat* genes and, to a lesser extent *asrABC,* suggested that some *Phycisphaeracaea* are capable of sulphate reduction via the siroheme-dependent anaerobic sulphite reductase (Figure S3; Anantharaman et al. 2018). Two genomes additionally contained the *dsrAB* genes. Between 40 and 60% of the SM1A02 family encoded either the *caa*_3_ or the *cbb*_3_-type cytochrome *c* oxidase (Figure S1). In 6 occasions, both oxidases were encoded and in 7 cases, a *bd*-type quinol oxidase was present. Both nitrate and sulfate respiration seemed to be phylogenetically more clustered than in *Phycisphaerales*, with several closely related genera encoding *narGH*, *nirK*, *norB*, and *nosZ*. For sulfate reduction, two distinct clusters of genera contained the *aprA* and *sat* genes. On the other hand, genes for sulphite reduction seemed to be absent in most of the genomes.

**Fig. 3:**
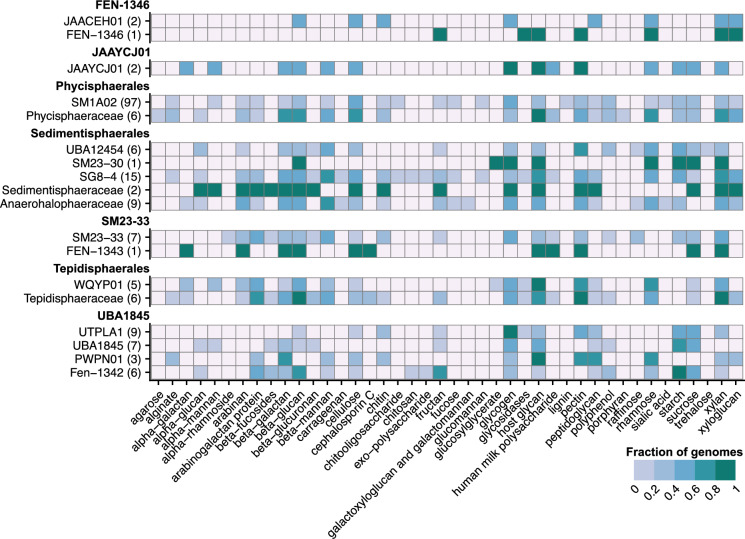
Substrate predictions by dbCAN4 for each family within the *Phycisphaerae*. The number behind each family represents the number of genomes in the analysis. Fill colour indicates the fraction of genomes in a family that are predicted to metabolize the substrate. Some substrate names represent groups of structurally similar compounds (listed in Table S2 and explained in detail in Zheng et al. 2023)

We were unable to find indications for a chemolithoautotrophic lifestyle in any *Phycisphaerales*, or, for that matter, *Phycisphaerae* genomes, as genes enabling the use of inorganic substrates (like, e.g., ammonium) as energy source or carbon dioxide fixation could not be identified. Therefore, we assume all members of the *Phycisphaerae*, including the *Phycisphaerales*, to be heterotrophic organisms using organic carbon as energy and carbon source. Most *Phycisphaerales* genomes contained a near-complete TCA cycle, with the observed incompleteness mainly due to a predicted lack of succinate dehydrogenase, which was often caused by a missing *sdhC* subunit annotation. Notably, the *Phycisphaeraceae* had a much higher CAZyme gene clusters (CGC) count than the SM1A02, indicating the potential for complex carbon compound degradation pathways. These CGCs were predicted to break down β-galactan, β-glucan, and host glycans (broad class of diverse glycans, derived from animals and plants), which further supports this hypothesis (Fig. 3).

### *Tepidisphaerales*

Of the included genomes, 11 were taxonomically assigned to the order *Tepidisphaerales*, mainly originating from anoxic terrestrial environments and sediments (Fig. 2) and distributed amongst the *Tepidisphaeraceae* and WQYP01 families. Three genera are described within the *Tepidisphaerales*: *Tepidisphaera, Humisphaera,* and *Fontivita*. However, only the genus *Tepidisphaera* was represented in our dataset. Most genomes of the *Tepidisphaerales* encode a partial ETC with NADH dehydrogenase, ACT, and the F-type ATPase genes being present. Additionally, some genomes also contain a *bd*-type quinol oxidase. Genes to utilize nitrate seem to be present in some genomes of the *Tepidisphaeraceae*. The *aprA*, *sat,* and *asrABC* genes are mostly found within the *Tepidisphaeraceae* and in some WQYP01, making it likely that *Tepidisphaerales* can use sulfate as an alternative electron acceptor. Two strains (SpSt − 394 and *T. mucosa*) encoded *ureABC*, enabling them to use urea as nitrogen source. All *Tepidisphaerales* contained a large number of CGCs, indicating their potential for complex carbon degradation. DBCan4 identified CGCs with arabinogalactans (e.g., β-galactan backbones with side chains of arabinan, rhamnose, or fucose), β-galactans, β-glucans, β-mannan, glycogen, host glycans, pectin, rhamnose, and xylan as potential substrates (Fig. 3).

### SM23-33

We obtained 8 genomes of the order SM23-33, originating from anoxic habitats. Most SM23-33 members completely lacked an ETC except for strain CAITIS01 (Fig. 2). This strain was found in an oxic freshwater environment and contained the NADH dehydrogenase, as well as ACT. Two other members (FEN-1343 and FEN-1344) also contained NADH dehydrogenase. The genomes of the strains CAITIS01, FEN-1343, and FEN-1344 contained a putative *norB* sequence, but respiration of inorganic nitrogen compounds was otherwise absent from the SM23-33 order. Sulphur reduction genes also were largely absent from genomes in the SM23-33 order. Several genomes showed HMM hits to *asrAB* and CAITIS01 may contain *asrABC* but sulphur respiration proteins were otherwise absent. The absence of respiratory capacity was also reflected in the incomplete annotation of the TCA cycle. Furthermore, SM23-33 genomes encoded relatively little CGCs and showed little conservation of the predicted substrates (Fig. 3). Nevertheless, xylan may be a common substrate utilized by this order.

### UBA1845

The UBA1845 order comprises the third-largest contribution to our dataset with 27 genomes passing the quality criteria. This order contained the FEN-1342 family with 68 ± 1 GC%, the highest of all *Phycisphaerae* (Fig. 1), but also the PWPN01 family, with a mean coding density of 85 ± 1%, the lowest of all *Phycisphaerae*. Genomes from this order were found in a variety of oxic and anoxic habitats, although mainly in engineered environments such as wastewater treatment, solid waste treatment, and lab-scale enrichments (Fig. 2). They contain mostly functional TCA cycles, which appear to be incomplete due to a missing succinate dehydrogenase annotation (see *Phycisphaerales*). Additionally, the pentose phosphate pathway (PPP) often appeared incomplete, which was due to a missing oxidative branch. Many genomes in this order have fewer CGCs than, for example, the *Tepidisphaerales* and *Sedimentisphaerales*, making it likely that this order is not involved in the breakdown of large or complex sugar molecules (Figs. 2 and 3). Still, CGCs responsible for the breakdown of starch and sucrose were predicted in most genomes. Most of the UBA1845 members had a complete annotation of the NADH dehydrogenase and ACT. On the other hand, only four genomes contained a full annotation of *bd*- or *cbb*_3_-type terminal oxidases. This seemed to be complemented by a widespread presence of *narGH* and *norBC* annotations. Surprisingly, nitrite reductases seemed to be lacking and only four genomes contained putative *nosZ* genes. However, hits with the hydroxylamine dehydrogenase (Hao) HMM on most UBA1845 revealed the presence of octaheme cytochrome *c* proteins related to nitrite reductase (Onr), responsible for dissimilatory nitrite reduction to nitric oxide or ammonium. Furthermore, *aprA*, *sat*, and *asrAB* seemed to be common in most UBA1845 genomes, making it likely that these are capable of nitrate, sulfate, and sulphite reduction.

### *Sedimentisphaerales*

The *Sedimentisphaerales* were the second largest genome set represented in this study, with 33 genomes retained for analysis. These genomes showed the lowest GC content of all *Phycisphaerae* with 44 ± 3% in the UBA12454 and 53 ± 3% in the *Anaerohalosphaeraceae* (Fig. 1). The latter family also contained the smallest genomes of the *Phycisphaerae* at 2.67 ± 0.80 Mb. This group appeared to be strictly fermentative with a very incomplete TCA cycle and a missing respiratory chain (Fig. 2). Within the *Sedimentisphaerales*, there is some difference between families with a higher (e.g., *Sedimentisphaeraceae*) and lower (e.g., *Anaerohalosphaeraceae*) CGC count, differentiating between the utilization of complex carbon substrates (Fig. 2). Xylan, rhamnose, pectin, glycogen, β-glucan, β-galactan, and arabinan degradation was commonly predicted in genomes of *Sedimentisphaerales* (Fig. 3). Most *Sedimentisphaerales* genomes also did not contain any genes for the reduction of inorganic nitrogen compounds. However, the families *Anaerohalosphaeraceae* and SG8-4 may be capable of nitrate and sulfate reduction.

### JAAYCJ01 and FEN-1346

The orders JAAYCJ01 and FEN-1346 were both severely underrepresented in our genome set with only two and three genomes included, respectively. Except for a single genome (JAAYCJ01), all genomes within these orders lacked an ETC and TCA cycle (Fig. 2). This suggests a strictly fermentative lifestyle, which gains further support from the absence of denitrification and sulfate reduction pathways in the FEN-1346 genomes. However, the JAAYCJ01 family encoded putative *aprA*, *sat*, and *asrAB* genes. To further confirm the taxonomic assignments of JAAYCJ01 and FEN-1346 as separate orders, we analysed the AAI within and between the *Phycisphaerae* orders (Figure S4). FEN-1346 alignments against other FEN-1346 genomes had a mean AAI of 56%. This was high in comparison to FEN-1346 *vs*. other *Phycisphaerae* genomes, which scored 45–53% AAI. Similarly, JAAYCJ01 shared 58% AAI, but only 45–53% AAI with other *Phycisphaerae* orders. Similar AAI scores were found within and between the other *Phycisphaerae* orders (Figure S4).

## Discussion

In the current research, we set out to characterize the metabolic potential encoded in the available medium- to high-quality *Phycisphaerae* genomes. Members of the *Phycisphaerae* have been found in a large variety of natural and engineered habitats but only few species have been brought into culture. Currently, the class *Phycisphaerae* is subdivided into the orders *Phycisphaerales*, *Sedimentisphaerales*, and *Tepidisphaerales*, as well as several unnamed orders without cultured representatives. These orders comprise nine isolated species, for five of which we included genomes in our analysis.

### *Phycisphaerales* are fresh- and saltwater inhabitants with the potential for symbiosis

The order *Phycisphaerales* contains four cultured representatives which are all part of the family *Phycisphaeraceae*. Isolated members of this family are *A. agarilytica* (Yoon et al. 2014), *M. calidilacus* (Kallscheuer et al. 2022), *P. mikurensis* (Fukunaga & Kurahashi 2009), and *Po. corsica* (Kallscheuer et al. 2020)*.* All described members of this family are heterotrophic, mesophilic, and able to respire oxygen. In addition, *P. mikurensis* has been shown to reduce nitrate under anaerobic conditions and the hydrolysis of agar, gelatin, and starch have been observed in this family. In our dataset, the *Phycisphaerales* comprised only a small fraction of the *Phycisphaeraceae* genomes. In line with previous observations, we found that the *Phycisphaerales* originated from oxic marine habitats and had the apparent ability to use oxygen as terminal electron acceptor. This family also contained the highest number of CGCs, supporting their involvement in the breakdown of complex carbon compounds. The characterized strains originate from marine algae (*P. mikurensis* and *A. agarilytica*), sponges (*Po. corsica*), and biofilms (*M. calidilacus*). The breakdown of complex carbon compounds secreted by eukaryotic partners could therefore be coupled to respiration and define the habitat for these *Phycisphaerae*. *Phycisphaeraceae* have also been detected in oxygen-minimum zones and suggested to be important in nitrogen cycling (Jasmin et al. 2017), and anoxic sediments, where they have been implicated with iron- (Feammox) or sulfate-dependent (Sulfammox) anaerobic ammonium oxidation (Rios-Del Toro et al. 2018). In our analysis, we found that most *Phycisphaeraceae* contained partial denitrification pathways to reduce nitrate to nitrous oxide. We also found that several genomes contained octaheme cytochrome *c* sequences similar to *hao*, which may indicate the presence of a dissimilatory octaheme cytochrome *c* nitrite reductase (Onr). This putative Onr may replace the function of nitrite reductase in *Phycisphaerae* lacking NirK and NirS. *Po. corsica* also encodes this octaheme cytochrome *c*, and thus the function of this protein could be tested in this pure culture. However, we could not find genes involved in (anaerobic) ammonia/ammonium oxidation in any of the genomes analysed. Therefore, it seems unlikely that *Phycisphaeraceae* are involved the oxidation of ammonium and instead make a living as facultative anaerobic complex carbon-degrading heterotrophs. Indeed, reduction of nitrate to nitrite and anaerobic growth on xylose were observed in *P. mikurensis* (Fukunaga and Kurahashi 2009)*.*

Most *Phycisphaerales* genomes were affiliated with the uncultured SM1A02 family, which have been found in metagenomes obtained from a wide variety of habitats. The family is split between representatives from marine and freshwater environments. Although the genomes affiliated with the SM1A02 contain a relatively small amount of CGCs, they may be involved in simple hydrocarbon degradation. For example, a previous study has found that members of SM1A02 can be abundant in crude oil-degrading enrichments (Uribe-Flores et al. 2019) and they have also been detected around hydrothermal vent systems (Storesund et al. 2018). Interestingly, one phylogenetic group affiliated with the genus GCA-002718515 showed poor completeness scores for central metabolic pathways such as the TCA cycle and PPP and contained the lowest number of CGCs. The genomes of this group were on average small (1.9 ± 0.07 Mb) and had a high coding density (94.1 ± 0.4%) compared to other SM1A02 genomes (3.0 ± 0.7 Mb, 91.7 ± 2.2%). Of the eight sequenced genomes in this genus, three were obtained from a hypersaline environment and one was obtained from a hydrothermal vent. Another two genomes were derived from metagenomic analysis of the glass sponge *Vazella pourtalesii* (Bayer et al. 2020). Here, it was also observed that the organisms recovered from the sponge microbiome had a relatively small genome size and may rely on nutrients provided by other organisms. Therefore, it appears that this genus of the SM1A02 is adapted to specific (extreme) environments and may depend on external amino acids for its survival.

### Facultatively anaerobic soil dwellers represent the *Tepidisphaerales*

The order *Tepidisphaerales* contains three isolates that are part of the *Tepidisphaeraceae* family, *F. pretiosa*, *H. borealis*, and *T. mucosa*. In contrast to what the name suggests, the *Tepidisphaerales* have been found both in hot springs and boreal peatlands. The *Tepidisphaerales* were originally characterized as the WD2101 ‘soil group’ (Dedysh et al. 2020) because they were often found in terrestrial environments. Indeed, *Tepidisphaerales* were detected in soils (Spring et al. 2018), peatland (Ivanova et al. 2016), and arctic rhizosphere (Parada-Pozo et al. 2022). *F. pretiosa* and *H. borealis* have also been shown to grow on plant polymers such as xylan (Kublanov 2022; Naumoff et al. 2022). While *T. mucosa* did not grow on xylan, it could degrade a range of other complex carbon substrates (Kovaleva et al. 2015). We also found a high number of CGCs in the *Tepidisphaerales* genomes, further highlighting this potential for complex carbon degradation. Under anaerobic conditions, *T. mucosa* was capable of fermentation and did not utilize inorganic nitrogen or sulphur compounds as electron acceptors (Kovaleva et al. 2015). Although we could confirm the absence of genes involved in denitrification, we did predict the presence of dissimilatory sulfate reduction in *T. mucosa* and, additionally, sulphite reduction in other *Tepidisphaeraceae*. The use of oxidized sulphur compounds by members of the *Tepidisphaeraceae*, however, still needs to be experimentally validated.

### Anaerobic respiration may complement fermentation in the SM23-33

The SM23-33 order currently comprises two families (SM23-33 and FEN-1343) of strictly fermentative *Phycisphaerae* bacteria. Literature descriptions of this order are scarce but they have been found in metagenomic datasets obtained from estuary sediments (Baker et al. 2015), sulphur-rich hydrothermal sediments (Zhou et al. 2020), and anaerobic digesters (Campanaro et al. 2020). We were unable to find any genes involved in (anaerobic) respiration, which supports a lifestyle adapted to electron acceptor-devoid environments. They also contained relatively high numbers of CGCs, which may extend their fermentative capacity to complex carbon substrates found in sediments. On the other hand, one single genome (CAITIS01, SM23-33 family) contained a putative NADH dehydrogenase, ACT, and *asrABC* required for sulphite reduction. This indicates that the diversity of the SM23-33 may currently be underestimated, and some SM23-33 members are capable of anaerobic sulphite reduction.

### Members of the UBA1845 order are often found in autotrophic enrichments

Bacteria affiliated with the order UBA1845 also are scarcely described in environmental studies and have only been encountered in larger metagenomic datasets. These metagenomes generally originate from anoxic environments such as hydrothermal sulphur-rich sediments (Zhou et al. 2020), anaerobic digesters (Campanaro et al. 2020), deep terrestrial subsurface fluids (Momper et al. 2017), thermal spring water (Pedron et al. 2019), and an anammox enrichment cultures (Zhao et al. 2018b). The UBA1845 also comprised our in-house genomes DAMOX_Bav1, TNR_N3, TNR_N4, TNR_A2, AOM_BES5, and COM_RAS2, which, except for TNR_A2, were obtained from low-oxygen or oxygen-depleted environments. Adaptation to low-oxygen environments was also supported by the presence of sulfate reduction and denitrification pathways. However, the presence of this group in several enrichment systems for autotrophic bacteria is curious (e.g., nitrifier and anammox enrichments where no organic carbon source is provided via the medium). It was recently suggested that some UBA1845 genomes contained *hzsABC*-like and *hao* genes required for anammox (Suarez et al. 2023). However, we were unable to confirm this using the HMM set for anammox genes included in Metascan (Cremers et al. 2022). We did find a putative *hao*-like octaheme cytochrome *c* sequence, which could be the Onr, involved in nitrite reduction to nitric oxide. The presence of Onr in the UTPLA1 and UBA1845 families would make sense, since most genomes encode incomplete denitrification pathways that miss *nirK* and *nirS* genes. The presence of *Phycisphaerae* in anammox enrichment cultures has been observed before and it has been suggested that they are capable of breaking down extracellular polysaccharides produced by anammox (Lawson et al. 2017; Zhao et al. 2018a).

### The *Sedimentisphaerales* are strictly fermentative

The order *Sedimentisphaerales* is subdivided into two families with isolated members, the *Anaerohalosphaeraceae* and the *Sedimentisphaeraceae*. The *Anaerohalosphaeraceae* contains a single cultivated species, *Anaerohalosphaera lusitana*, which is strictly fermentative with sugars as preferred substrates (Pradel et al. 2020). The *Sedimentisphaeraceae* comprises three isolates, *Limihaloglobus sulfuriphilus* (Pradel et al. 2020), *Sedimentisphaera cyanobacteriorum* (Spring et al. 2018)*,* and *Sedimentisphaera salicampi* (Spring et al. 2018). Both genera are strictly fermentative and the *Sedimentisphaera* appear capable of breaking down more polysaccharides than the *Anaerohalosphaera*. This was also reflected in the number of CGCs we found in both families, which was substantially higher in the *Sedimentisphaera* but also in the SG8-4 and some members of the UBA12454 families. A strictly fermentative lifestyle was also found for the uncultured *Sedimentisphaerales*, denoted by the absence of an ETC and TCA cycle. So far, genomes affiliated with the *Sedimentisphaerales* have been found in anoxic aquatic sediments (Baker et al. 2015; Dombrowski et al. 2017; Spring et al. 2018; Zhou et al. 2020), terrestrial sediments (Hernsdorf et al. 2017), anaerobic digesters (Campanaro et al. 2020), and an aquifer where oxygen may still be present (Anantharaman et al. 2016). Here, we found indications that most *Anaerohalosphaeraceae* are capable of nitrate, sulfate, and sulphite reduction but only sulfate assimilation was verified experimentally in *A. lusitana* and nitrate could not be reduced under the tested conditions (Pradel et al. 2020).

### JAAYCJ01 and FEN-1346

Members of the orders JAAYCJ01 and FEN-1346 have not been described before in the literature and are rarely found in metagenomic datasets. Of the genomes included in our dataset, one (FEN-1346) was derived from dimethylsulfoniopropionate-contaminated sediment (Song et al. 2020) and three (FEN-1346 and JAAYCJ01) were obtained from an anaerobic digester (Campanaro et al. 2020). Other genomes that were included in this study originated from permafrost and freshwater environments. Finally, a FEN-1346-affiliated genome was also obtained from 20,000 to 1,000,000-year-old permafrost soil (Sipes et al. 2021) but was not taken into analysis. The few available genomes give the impression that the FEN-1346 are strictly fermentative organisms while the JAAYCJ01 order may also include members with (anaerobic) respiration capabilities, but it remains difficult to speculate on their lifestyles with the limited amount of information available. Nonetheless, the AAI values of JAAYCJ01 and FEN-1346 compared to other orders in the *Phycisphaerae* confirmed that these are separate orders. The lack of genomes within these orders may therefore represent a wealth of undiscovered phylogenetic diversity within this class.

### Microbial garbagemen

Bacterial members of the class *Phycisphaerae* occur in many genomic datasets obtained from a wide variety of environments. Despite their ubiquitous distribution, the *Phycisphaerae* have eluded culturing attempts, resulting in the availability of only few described species. Nevertheless, several reports report enrichment of *Phycisphaerae*, for example, to 17.7% in cultures enriched on sugars (Cui et al. 2016), 10% in a nitrate-dependent anaerobic methane oxidation and anammox enrichment (Stultiens et al. 2020), and 24.5% in an anammox enrichment (Zhao et al. 2018a). Such enrichment of a heterotrophic side-community is interesting, considering that the latter 3 systems were operated without addition of organic carbon, but has been observed before in anammox cultures where *Chloroflexota* comprised up to 10% of the community (Kindaichi et al. 2012). The authors found that these *Chloroflexota* incorporated decaying anammox cell material by employing FISH-MAR to establish whether ^14^C-radioisotopically labelled carbon substrates that might be produced by anammox bacteria were consumed by the side-community (Kindaichi et al. 2012). Similarly, we expect that many *Phycisphaerae* are capable of a ‘garbagemen’ lifestyle in such ecosystems. *Phycisphaerae* appear to be adapted to thrive on a wealth of different sugar and complex carbon substrates excreted by primary producers. In some *Phycisphaerae* families, this is signified by the high number of CGCs encoded in their genomes. Therefore, further enrichment and isolation attempts of the *Phycisphaerae* may focus on using a mixture of (complex) carbon substrates or utilizing the compounds that are excreted by primary producers. To give these attempts direction, availability of transcriptome data should help in elucidating the CGCs active in a complex community. Here, we provide an overview of putative carbon substrates that could aid in enrichment and isolation attempts. The common prediction of host glycans, xylan, glycogen, beta-galactans, and beta-glucans breakdown in *Phycisphaerae* indicates that cell debris from bacterial and eukaryotic communities may be a good substrate for multiple families of this class.

## Supplementary Information

Supplementary file 1

## Data Availability

The NCBI accession numbers of all genomes included in this study are provided in Table S1. Genomes that were obtained in-house are available at the references mentioned in the methods section.
