# Supplementary material for: Genomic analysis of the class Phycisphaerae reveals a versatile group of complex carbon-degrading bacteria
Source: Antonie Van Leeuwenhoek. 2024 Jul 23;117(1):104. doi: 10.1007/s10482-024-02002-7 (PMC11266412; doi:10.1007/s10482-024-02002-7)
Supplement: Supplementary file 1 [file 10482_2024_2002_MOESM1_ESM.pdf]

Genomic analysis of the class *Phycisphaerae* reveals a versatile group of complex carbon-degrading bacteria

Wouter B. Lenferink, Theo A. van Alen, Mike S.M. Jetten, Huub J.M. Op den Camp, Maartje A.H.J. van Kessel, Sebastian Lücker*

SUPPLEMENTARY INFORMATION

Table of contents

- Supplementary Figures S1 – S4
- Supplementary Tables S1 – S2

Supplementary Figures and Tables

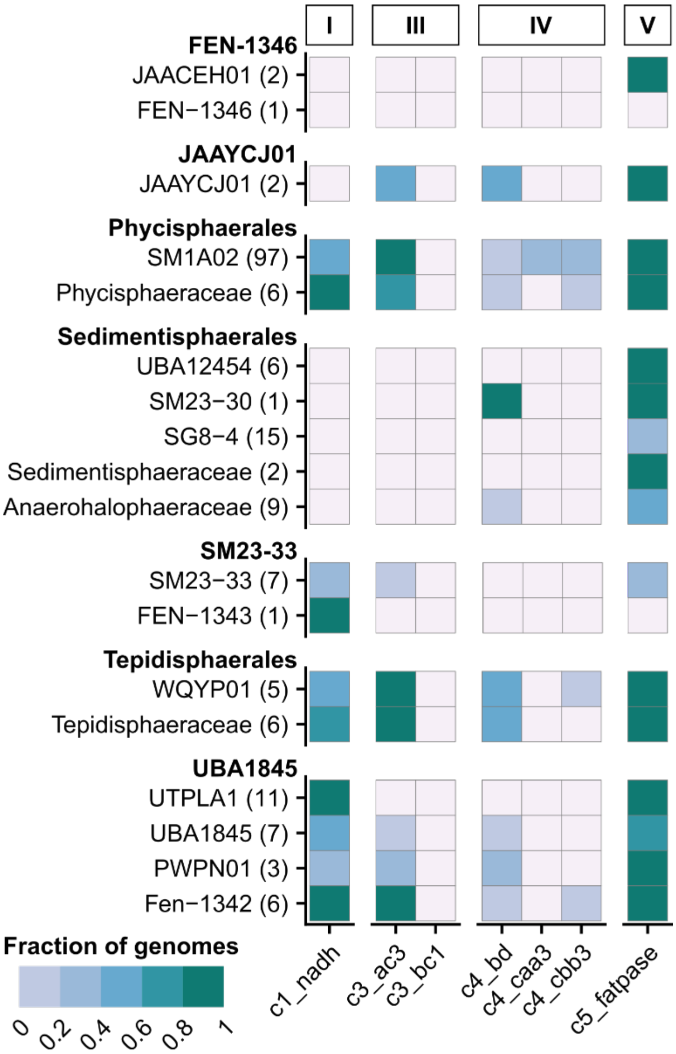

Supplementary Figure 1. Automatic and manually curated annotation of electron transfer chain (ETC) components, summarized for each *Phycisphaerae* family. From left to right, hits are displayed for the NADH dehydrogenase (c1_nadh), alternative complex III (c3_ac3), cytochrome *bc*$_1$ complex (c3_bc1), *bd*-type quinol oxidase (c4_bd), *caa*$_3$-type cytochrome *c* oxidase (c4_caa3), *cbb*$_3$-type cytochrome *c* oxidase (c4_cbb3), and the F-type ATPase (c5_fatpase). Numbers in parentheses behind family names represent the number of included genomes. Fill colour represents the fraction of genomes in a family predicted to encode the complete complex.

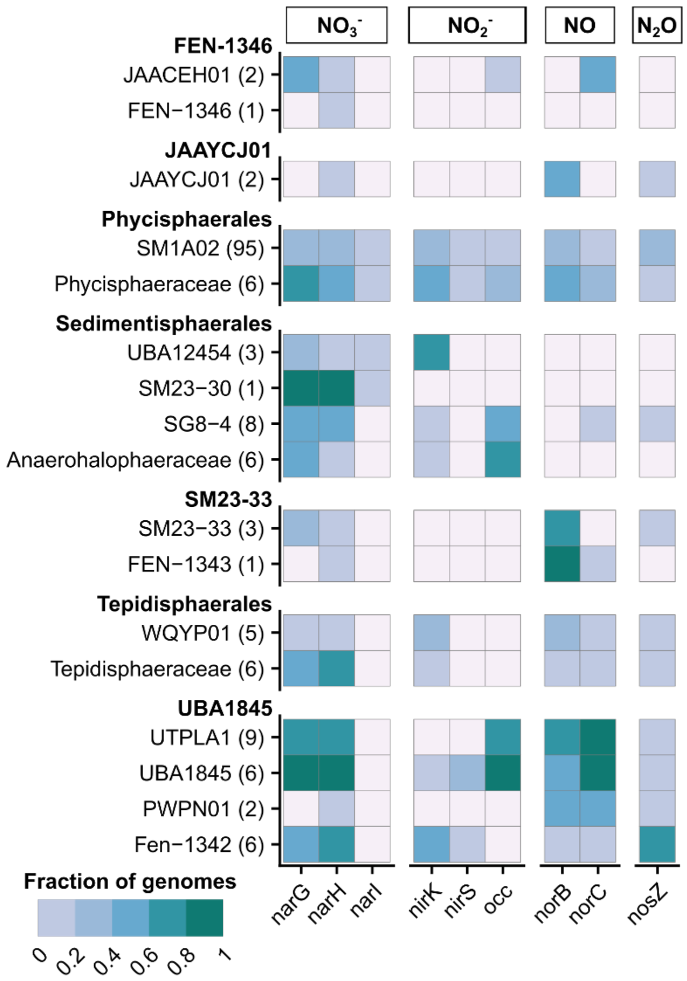

Supplementary Figure 2. Manually curated annotation of genes involved in denitrification, summarized for each *Phycisphaerae* family. From left to right, hits are displayed for nitrate (*narGHI*), nitrite (*nirK*, *nirS*, *occ*), nitric oxide (*norBC*), and nitrous oxide (*nosZ*) reduction. Numbers in parentheses behind family names represent the number of included genomes. Fill colours represent the fraction of genomes per family predicted to contain the respective gene.

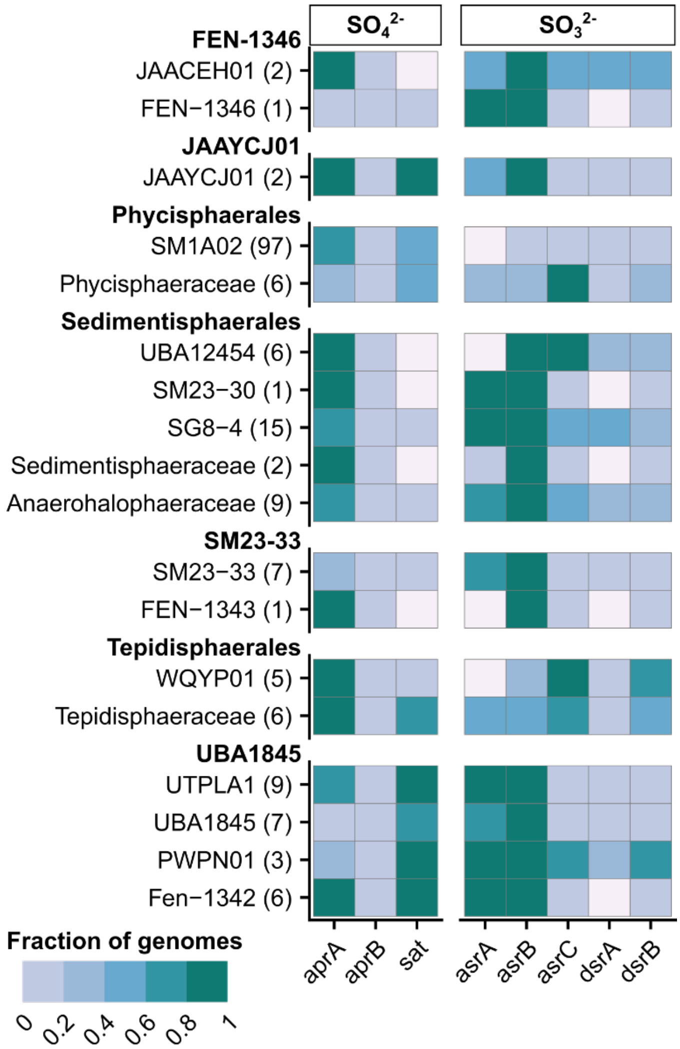

Supplementary Figure 3. Manually curated annotation of genes involved in sulphur reduction, summarized for each *Phycisphaerae* family. From left to right, hits are displayed for sulfate (*aprAB*, *sat*) and sulphite (*asrABC*, *dsrAB*) reduction. Numbers in parentheses behind family names represent the number of included genomes. Fill colours represent the fraction of genomes per family predicted to contain the respective gene.

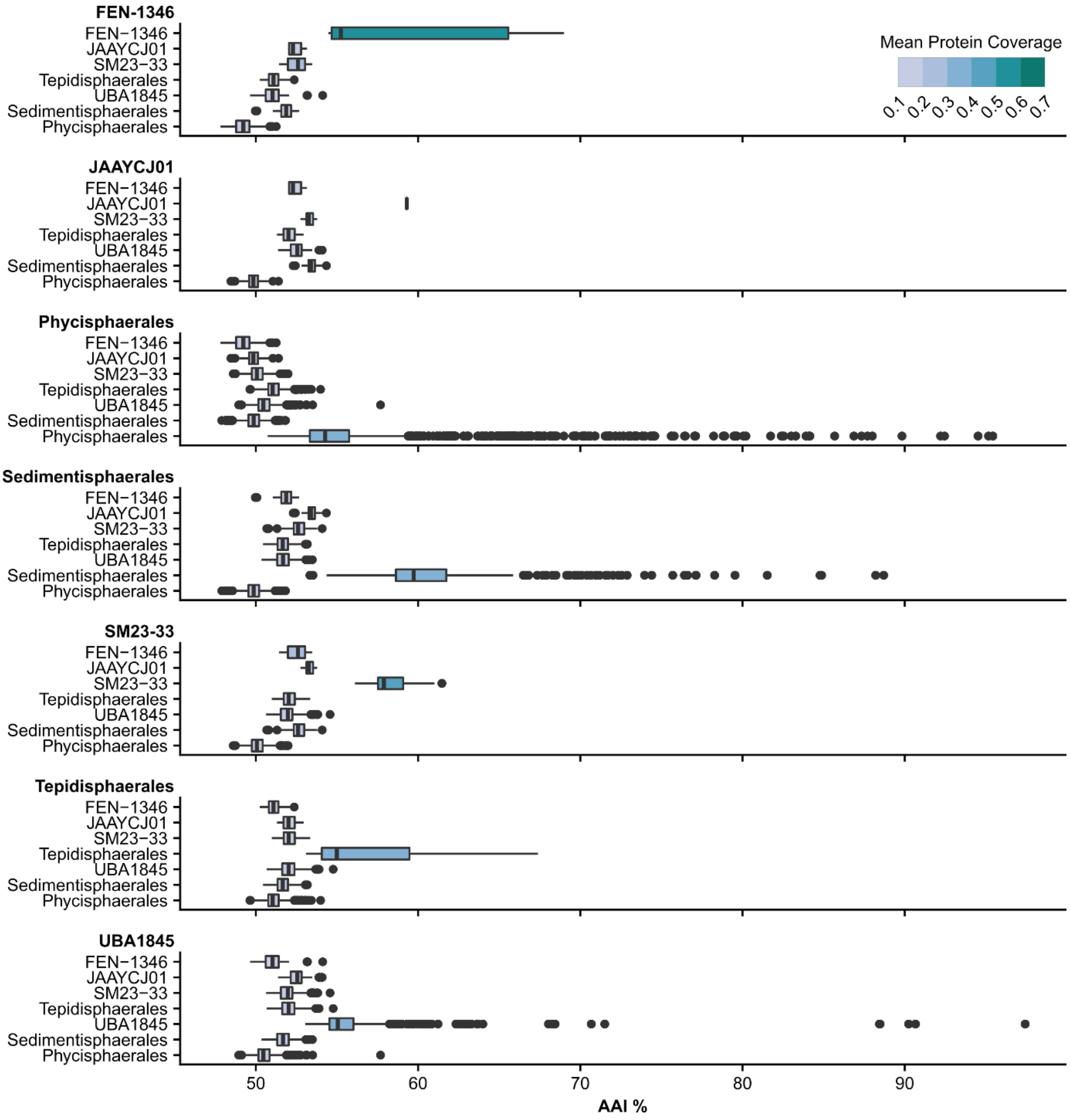

Supplementary Figure 4. Average amino acid identity (AAI) comparisons between distinct *Phycisphaerae* orders. Names in bold indicate the respective order all other genomes were compared to.

Table S1. Genome assemblies included in this study, corresponding CheckM statistics, and GTDB-Tk taxonomy. Asterisks indicate in-house genomes.

Assembly	Completeness	Contamination	Coding density	GC content	Size	Order	Family	Genus	Species
**AMOX_SL1***	86.8%	2.1%	87.9%	60.8%	4.90 Mb	Phycisphaerales	SM1A02	NA	AMOX_SL1
**AOM_BES5***	91.5%	1.6%	86.3%	65.6%	4.29 Mb	UBA1845	UBA1845	SURF-32	AOM_BES5
**AOM_Fe1***	94.7%	2.0%	89.3%	58.8%	5.28 Mb	Sedimentisphaerales	Anaerohalophaeraceae	B28-G16	AOM_Fe1
**COM_RAS1***	89.1%	1.6%	86.7%	66.1%	5.50 Mb	UBA1845	Fen-1342	NA	COM_RAS1
**COM_RAS2***	89.8%	0.8%	88.6%	62.6%	4.33 Mb	UBA1845	UTPLA1	PLA3	COM_RAS2
**COM_TrWB2***	83.7%	2.1%	87.1%	65.8%	4.20 Mb	Phycisphaerales	SM1A02	UBA2396	COM_TrWB2
**DAMOX_Bav1***	87.1%	0.4%	87.0%	60.6%	5.04 Mb	UBA1845	UTPLA1	UTPLA1	DAMOX_Bav1
**GCA_000284115.1**	95.5%	0.0%	88.5%	73.2%	3.88 Mb	Phycisphaerales	Phycisphaeraceae	Phycisphaera	Phycisphaera mikurensis
**GCA_001303465.1**	71.6%	0.0%	88.2%	54.8%	4.46 Mb	Sedimentisphaerales	SG8-4	SG8-4	SG8-4 sp001303465
**GCA_001303665.1**	75.0%	1.1%	88.5%	67.8%	3.43 Mb	SM23-33	SM23-33	SM23-33	SM23-33 sp001303665
**GCA_001303695.1**	70.8%	0.0%	87.5%	51.0%	4.01 Mb	Sedimentisphaerales	SM23-30	SM23-30	SM23-30 sp001303695
**GCA_001603075.1**	100.0%	1.1%	89.3%	62.8%	5.65 Mb	Sedimentisphaerales	SG8-4	PLanc-01	PLanc-01 sp001603075
**GCA_001824635.1**	87.3%	1.7%	86.3%	49.5%	2.62 Mb	Sedimentisphaerales	UBA12454	GWF2-50-10	GWF2-50-10 sp001824635
**GCA_001825665.1**	97.7%	1.1%	90.6%	41.0%	4.83 Mb	Sedimentisphaerales	UBA12454	UBA12454	UBA12454 sp001825665
**GCA_001825675.1**	92.1%	3.4%	88.6%	42.0%	4.86 Mb	Sedimentisphaerales	UBA12454	UBA12454	UBA12454 sp001825675
**GCA_001997385.1**	98.9%	1.1%	89.4%	46.2%	2.95 Mb	Sedimentisphaerales	Sedimentisphaeraceae	Sedimentisphaera	Sedimentisphaera cyanobacteriorum
**GCA_002084585.1**	89.8%	1.1%	87.9%	50.1%	1.81 Mb	Sedimentisphaerales	Anaerohalophaeraceae	4572-13	4572-13 sp002084585
**GCA_002117005.1**	98.9%	1.1%	89.5%	46.3%	3.19 Mb	Sedimentisphaerales	Sedimentisphaeraceae	Sedimentisphaera	Sedimentisphaera salicampi
**GCA_002167845.2**	93.1%	1.0%	91.1%	63.2%	3.31 Mb	Phycisphaerales	SM1A02	UBA12014	UBA12014 sp002167845
**GCA_002171655.1**	80.6%	1.7%	91.9%	58.7%	2.98 Mb	Phycisphaerales	SM1A02	UBA8087	UBA8087 sp002171655
**GCA_002295885.1**	96.0%	1.1%	91.8%	65.1%	2.34 Mb	Phycisphaerales	SM1A02	UBA854	UBA854 sp002295885
**GCA_002311745.1**	79.5%	0.0%	89.5%	62.7%	2.93 Mb	Tepidisphaerales	Tepidisphaeraceae	UBA1161	UBA1161 sp002311745
**GCA_002327785.1**	89.3%	5.4%	87.1%	60.8%	6.32 Mb	Sedimentisphaerales	SG8-4	PLanc-01	PLanc-01 sp002327785

Table S1. Continued.

**GCA_002337545.1**	88.5%	0.0%	87.5%	59.0%	3.53 Mb	Phycisphaerales	SM1A02	UBA1924	UBA1924 sp002337545
**GCA_002338715.1**	91.4%	3.4%	85.6%	57.0%	5.25 Mb	UBA1845	UBA1845	UBA1845	UBA1845 sp002338715
**GCA_002343075.1**	92.2%	3.4%	87.5%	59.6%	4.47 Mb	Tepidisphaerales	Tepidisphaeraceae	UBA2421	UBA2421 sp002343075
**GCA_002343325.1**	81.7%	1.1%	89.0%	67.2%	3.13 Mb	Phycisphaerales	SM1A02	UBA2396	UBA2396 sp002343325
**GCA_002347125.1**	87.3%	4.6%	88.8%	62.3%	4.76 Mb	Sedimentisphaerales	SG8-4	UBA2266	UBA2266 sp002347125
**GCA_002364005.1**	91.5%	1.1%	88.0%	61.7%	3.40 Mb	Phycisphaerales	SM1A02	UBA1924	UBA1924 sp002364005
**GCA_002364485.1**	97.4%	1.1%	92.0%	62.9%	2.56 Mb	Phycisphaerales	SM1A02	UBA854	UBA854 sp002364485
**GCA_002364555.1**	96.7%	0.6%	90.4%	57.4%	2.59 Mb	Phycisphaerales	SM1A02	UBA1668	UBA1668 sp002364555
**GCA_002418285.1**	96.6%	0.0%	89.9%	61.9%	4.84 Mb	Phycisphaerales	SM1A02	UBA5793	UBA5793 sp002418285
**GCA_002480345.1**	91.4%	0.0%	86.8%	67.9%	3.85 Mb	Phycisphaerales	SM1A02	UBA1926	UBA1926 sp002480345
**GCA_002480565.1**	95.3%	0.0%	88.7%	50.0%	6.01 Mb	Phycisphaerales	Phycisphaeraceae	UBA7800	UBA7800 sp002480565
**GCA_002591705.1**	82.7%	4.2%	91.9%	59.6%	2.53 Mb	Phycisphaerales	SM1A02	UBA854	UBA854 sp002591705
**GCA_002683195.1**	89.0%	1.7%	91.0%	63.4%	3.59 Mb	Phycisphaerales	SM1A02	GCA-002862325	GCA-002862325 sp002683195
**GCA_002684315.1**	74.5%	4.6%	92.8%	59.2%	2.73 Mb	Phycisphaerales	SM1A02	UBA8087	UBA8087 sp002684315
**GCA_002684755.1**	83.3%	5.1%	91.0%	66.7%	2.98 Mb	Phycisphaerales	SM1A02	UBA12014	UBA12014 sp002684755
**GCA_002686995.1**	80.7%	0.6%	90.4%	66.5%	7.80 Mb	Phycisphaerales	Phycisphaeraceae	GCA-2686995	GCA-2686995 sp002686995
**GCA_002689385.1**	90.9%	3.4%	93.6%	45.1%	1.94 Mb	Phycisphaerales	SM1A02	GCA-002718515	GCA-002718515 sp002689385
**GCA_002690755.1**	94.3%	4.1%	90.5%	66.1%	3.47 Mb	Phycisphaerales	SM1A02	UBA12014	UBA12014 sp002690755
**GCA_002700845.1**	76.9%	4.0%	94.1%	55.1%	1.77 Mb	Phycisphaerales	SM1A02	UBA8653	UBA8653 sp002700845
**GCA_002702575.1**	98.9%	6.3%	93.5%	57.8%	3.17 Mb	Phycisphaerales	SM1A02	UBA1668	UBA1668 sp002702575
**GCA_002704825.1**	83.1%	3.5%	88.8%	51.8%	6.42 Mb	Phycisphaerales	Phycisphaeraceae	UBA7800	UBA7800 sp002704825
**GCA_002706885.1**	85.2%	0.3%	90.6%	65.9%	3.57 Mb	Phycisphaerales	SM1A02	GCA-2706885	GCA-2706885 sp002706885
**GCA_002709295.1**	98.9%	4.0%	91.9%	65.7%	3.68 Mb	Phycisphaerales	SM1A02	GCA-002862325	GCA-002862325 sp002709295
**GCA_002709755.1**	94.3%	0.6%	92.4%	59.9%	2.62 Mb	Phycisphaerales	SM1A02	UBA8087	UBA8087 sp002709755
**GCA_002711415.1**	96.6%	0.6%	93.0%	61.0%	2.80 Mb	Phycisphaerales	SM1A02	GCA-2711415	GCA-2711415 sp002711415
**GCA_002716385.1**	84.7%	6.6%	93.4%	57.7%	2.79 Mb	Phycisphaerales	SM1A02	UBA1668	UBA1668 sp002716385
**GCA_002716625.1**	89.5%	0.0%	89.3%	50.9%	3.35 Mb	Phycisphaerales	Phycisphaeraceae	UBA6626	UBA6626 sp002716625
**GCA_002717655.1**	93.1%	1.8%	93.3%	55.2%	3.07 Mb	Phycisphaerales	SM1A02	UBA1668	UBA1668 sp002717655

Table S1. Continued.

**GCA_002718515.1**	85.5%	6.3%	94.8%	45.3%	1.95 Mb	Phycisphaerales	SM1A02	GCA-002718515	GCA-002718515 sp002718515
**GCA_002839495.1**	88.1%	3.4%	89.0%	45.5%	1.83 Mb	Sedimentisphaerales	UBA12454	UBA12454	UBA12454 sp002839495
**GCA_002862305.1**	97.7%	2.3%	92.3%	68.0%	2.67 Mb	Phycisphaerales	SM1A02	UBA8087	UBA8087 sp002862305
**GCA_002862325.1**	82.6%	1.8%	91.9%	64.9%	2.44 Mb	Phycisphaerales	SM1A02	GCA-002862325	GCA-002862325 sp002862325
**GCA_003141275.1**	96.0%	3.5%	88.6%	49.2%	3.05 Mb	Sedimentisphaerales	SG8-4	Fen-1362	Fen-1362 sp003141275
**GCA_003141315.1**	96.6%	1.1%	88.7%	47.7%	2.35 Mb	Sedimentisphaerales	SG8-4	Fen-1359	Fen-1359 sp003141315
**GCA_003142595.1**	97.7%	1.1%	90.3%	44.0%	2.28 Mb	Sedimentisphaerales	UBA12454	UBA12454	UBA12454 sp003142595
**GCA_003153655.1**	89.6%	2.3%	89.4%	57.0%	4.65 Mb	Tepidisphaerales	Tepidisphaeraceae	PALSA-1353	PALSA-1353 sp003153655
**GCA_003153915.1**	79.8%	2.3%	88.1%	62.4%	2.63 Mb	SM23-33	SM23-33	FEN-1349	FEN-1349 sp003153915
**GCA_003162245.1**	94.7%	6.8%	87.6%	67.1%	4.76 Mb	UBA1845	Fen-1342	Fen-1342	Fen-1342 sp003162245
**GCA_003170085.1**	89.5%	0.9%	87.8%	66.0%	4.47 Mb	FEN-1346	FEN-1346	FEN-1346	FEN-1346 sp003170085
**GCA_003170415.1**	96.6%	1.1%	88.7%	49.8%	2.67 Mb	Sedimentisphaerales	SG8-4	Fen-1362	Fen-1362 sp003170415
**GCA_003171235.1**	87.2%	1.1%	84.2%	64.2%	3.61 Mb	SM23-33	SM23-33	FEN-1344	FEN-1344 sp003171235
**GCA_003171335.1**	95.5%	1.1%	84.3%	64.4%	4.20 Mb	SM23-33	FEN-1343	FEN-1343	FEN-1343 sp003171335
**GCA_003520025.1**	82.9%	0.0%	89.8%	60.5%	2.13 Mb	Phycisphaerales	SM1A02	UBA12567	UBA12567 sp003520025
**GCA_003551645.1**	95.5%	2.3%	87.7%	63.5%	2.83 Mb	SM23-33	SM23-33	PUND01	PUND01 sp003551645
**GCA_003562195.1**	89.2%	4.0%	91.2%	57.8%	2.75 Mb	Phycisphaerales	SM1A02	PWVT01	PWVT01 sp003562195
**GCA_003576875.1**	89.8%	0.0%	89.3%	63.5%	3.36 Mb	UBA1845	UTPLA1	PLA3	PLA3 sp003576875
**GCA_003576905.1**	97.7%	3.4%	88.1%	63.8%	4.71 Mb	UBA1845	UTPLA1	UTPLA1	UTPLA1 sp003576905
**GCA_003597935.1**	72.7%	4.0%	84.8%	67.3%	4.30 Mb	UBA1845	UBA1845	SURF-32	SURF-32 sp003597935
**GCA_003644175.1**	82.7%	1.2%	88.3%	53.6%	2.51 Mb	Sedimentisphaerales	Anaerohalophaeraceae	B28-G16	B28-G16 sp003644175
**GCA_003644205.1**	77.8%	1.7%	89.1%	48.7%	2.64 Mb	Sedimentisphaerales	Anaerohalophaeraceae	QNBT01	QNBT01 sp003644205
**GCA_003644295.1**	77.1%	3.9%	89.5%	45.7%	1.82 Mb	Sedimentisphaerales	SG8-4	B140-G9	B140-G9 sp003644295
**GCA_003671025.1**	95.5%	0.6%	90.4%	60.3%	3.25 Mb	Phycisphaerales	SM1A02	QWPT01	QWPT01 sp003671025
**GCA_003671055.1**	96.6%	2.8%	92.1%	58.2%	2.85 Mb	Phycisphaerales	SM1A02	F1-140-MAGs142	F1-140-MAGs142 sp003671055
**GCA_003671065.1**	76.1%	0.0%	92.7%	58.5%	2.45 Mb	Phycisphaerales	SM1A02	QWPT01	QWPT01 sp003671065
**GCA_003671075.1**	93.2%	0.6%	91.0%	63.0%	3.35 Mb	Phycisphaerales	SM1A02	QWPT01	QWPT01 sp003671075
**GCA_003696675.1**	95.5%	2.3%	90.7%	66.0%	3.28 Mb	Phycisphaerales	SM1A02	UBA6054	UBA6054 sp003696675

Table S1. Continued.

**GCA_003696725.1**	95.5%	1.7%	92.0%	72.4%	2.46 Mb	Phycisphaerales	SM1A02	J020	J020 sp003696725
**GCA_003697045.1**	94.9%	1.1%	92.4%	62.3%	2.83 Mb	Phycisphaerales	SM1A02	J022	J022 sp003697045
**GCA_005223045.1**	94.3%	2.3%	88.1%	52.9%	5.98 Mb	Sedimentisphaerales	SG8-4	SG8-4	SG8-4 sp005223045
**GCA_005240095.1**	89.7%	0.6%	88.9%	63.6%	4.67 Mb	UBA1845	UBA1845	SBAX01	SBAX01 sp005240095
**GCA_005776885.1**	86.0%	0.6%	93.8%	66.6%	2.49 Mb	Phycisphaerales	SM1A02	SXOD01	SXOD01 sp005776885
**GCA_005787685.1**	95.3%	1.1%	92.9%	53.3%	2.37 Mb	Phycisphaerales	SM1A02	SYAC01	SYAC01 sp005787685
**GCA_005789005.1**	82.3%	1.2%	93.0%	53.7%	2.23 Mb	Phycisphaerales	SM1A02	SYAC01	SYAC01 sp005789005
**GCA_005790565.1**	91.3%	0.6%	92.8%	60.6%	2.66 Mb	Phycisphaerales	SM1A02	F1-140-MAGs142	F1-140-MAGs142 sp005790565
**GCA_005790605.1**	91.6%	0.6%	92.7%	59.9%	3.03 Mb	Phycisphaerales	SM1A02	F1-140-MAGs142	F1-140-MAGs142 sp005790605
**GCA_005792145.1**	98.5%	0.0%	94.4%	52.0%	2.52 Mb	Phycisphaerales	SM1A02	F1-60-MAGs104	F1-60-MAGs104 sp005792145
**GCA_005798965.1**	75.1%	0.6%	94.3%	67.0%	2.06 Mb	Phycisphaerales	SM1A02	SYIB01	SYIB01 sp005798965
**GCA_005799205.1**	79.0%	0.0%	96.1%	60.0%	2.0 Mb	Phycisphaerales	SM1A02	F1-60-MAGs104	F1-60-MAGs104 sp005799205
**GCA_005799475.1**	88.4%	0.6%	95.6%	65.1%	2.17 Mb	Phycisphaerales	SM1A02	F1-60-MAGs104	F1-60-MAGs104 sp005799475
**GCA_005799495.1**	96.0%	1.1%	94.8%	63.5%	2.36 Mb	Phycisphaerales	SM1A02	F1-60-MAGs104	F1-60-MAGs104 sp005799495
**GCA_006969385.1**	98.9%	0.0%	86.3%	57.3%	5.48 Mb	UBA1845	UTPLA1	ECT2AJA-110-A	ECT2AJA-110-A sp006969385
**GCA_007121565.1**	79.5%	1.2%	88.9%	57.3%	1.72 Mb	Sedimentisphaerales	Anaerohalophaeraceae	T3Sed10-213	T3Sed10-213 sp007121565
**GCA_007124345.1**	73.9%	2.7%	90.0%	67.2%	2.87 Mb	Phycisphaerales	SM1A02	SLFH01	SLFH01 sp007124345
**GCA_007693725.1**	92.1%	0.0%	91.1%	64.8%	2.71 Mb	Phycisphaerales	SM1A02	REDD01	REDD01 sp007693725
**GCA_007693765.1**	93.3%	0.0%	88.7%	69.7%	2.90 Mb	Phycisphaerales	SM1A02	RECZ01	RECZ01 sp007693765
**GCA_007693785.1**	95.5%	1.1%	88.2%	69.4%	3.06 Mb	Phycisphaerales	SM1A02	RECY01	RECY01 sp007693785
**GCA_007693845.1**	94.0%	4.0%	88.5%	61.3%	3.26 Mb	Phycisphaerales	SM1A02	SKZB01	SKZB01 sp007693845
**GCA_007693965.1**	97.7%	0.0%	87.9%	65.8%	3.48 Mb	Phycisphaerales	SM1A02	RECQ01	RECQ01 sp007693965
**GCA_007694925.1**	91.5%	0.0%	90.0%	68.1%	2.99 Mb	Phycisphaerales	SM1A02	SLFH01	SLFH01 sp007694925
**GCA_007747445.1**	94.3%	0.0%	84.1%	48.7%	4.29 Mb	Phycisphaerales	Phycisphaeraceae	Poriferasphaera	Poriferasphaera corsicensis
**GCA_008363215.1**	96.6%	0.0%	89.1%	65.8%	3.57 Mb	Phycisphaerales	SM1A02	J022	J022 sp008363215
**GCA_009691705.1**	97.4%	1.1%	95.4%	58.7%	1.99 Mb	Phycisphaerales	SM1A02	F1-60-MAGs104	F1-60-MAGs104 sp009691705
**GCA_009691725.1**	95.1%	0.0%	95.3%	58.5%	2.07 Mb	Phycisphaerales	SM1A02	F1-60-MAGs104	F1-60-MAGs104 sp009691725
**GCA_009691805.1**	92.1%	0.6%	92.8%	58.6%	2.62 Mb	Phycisphaerales	SM1A02	F1-140-MAGs142	F1-140-MAGs142 sp009691805

Table S1. Continued.

**GCA_009691915.1**	96.2%	0.0%	95.2%	63.6%	2.27 Mb	Phycisphaerales	SM1A02	F1-60-MAGs104	F1-60-MAGs104 sp009691915
**GCA_009692015.1**	88.3%	0.0%	95.5%	60.4%	1.74 Mb	Phycisphaerales	SM1A02	F1-60-MAGs104	F1-60-MAGs104 sp009692015
**GCA_009692045.1**	96.2%	1.1%	94.9%	60.3%	2.17 Mb	Phycisphaerales	SM1A02	SYPI01	SYPI01 sp009692045
**GCA_009781185.1**	91.5%	1.1%	87.2%	57.0%	4.65 Mb	Tepidisphaerales	WQYP01	WQYP01	WQYP01 sp009781185
**GCA_009827205.1**	89.7%	2.8%	92.5%	60.4%	2.94 Mb	Phycisphaerales	SM1A02	GCA-2732755	GCA-2732755 sp009827205
**GCA_009827215.1**	89.5%	0.0%	92.0%	56.2%	2.59 Mb	Phycisphaerales	SM1A02	UBA1924	UBA1924 sp009827215
**GCA_009908915.1**	80.1%	1.1%	85.7%	64.3%	2.95 Mb	SM23-33	SM23-33	JAAAOJ01	JAAAOJ01 sp009908915
**GCA_009926625.1**	98.5%	1.1%	94.2%	63.6%	2.58 Mb	Phycisphaerales	SM1A02	F1-60-MAGs104	F1-60-MAGs104 sp009926625
**GCA_009926815.1**	76.5%	4.0%	94.2%	69.5%	2.29 Mb	Phycisphaerales	SM1A02	SYAC01	SYAC01 sp009926815
**GCA_011048645.1**	88.6%	0.1%	90.0%	55.3%	2.49 Mb	Sedimentisphaerales	Anaerohalophaeraceae	T3Sed10-213	T3Sed10-213 sp011048645
**GCA_011054455.1**	86.9%	0.5%	87.6%	68.9%	3.97 Mb	UBA1845	Fen-1342	DSPM01	DSPM01 sp011054455
**GCA_011332905.1**	97.7%	1.1%	93.3%	55.7%	2.87 Mb	Sedimentisphaerales	SG8-4	DTPU01	DTPU01 sp011332905
**GCA_011367345.1**	96.6%	0.0%	91.4%	67.1%	4.28 Mb	Tepidisphaerales	Tepidisphaeraceae	DSYW01	DSYW01 sp011367345
**GCA_011369955.1**	100.0%	1.1%	90.4%	67.0%	3.88 Mb	UBA1845	UTPLA1	SpSt-539	SpSt-539 sp011369955
**GCA_011370245.1**	96.6%	2.3%	90.3%	61.4%	5.15 Mb	Tepidisphaerales	WQYP01	SpSt-394	SpSt-394 sp011370245
**GCA_011525745.1**	97.7%	1.1%	83.4%	65.3%	5.40 Mb	UBA1845	UTPLA1	PLA4	PLA4 sp011525745
**GCA_011525805.1**	95.5%	0.0%	88.2%	67.0%	4.54 Mb	Phycisphaerales	SM1A02	UBA5793	UBA5793 sp011525805
**GCA_011682595.1**	75.8%	1.1%	87.5%	64.4%	2.43 Mb	FEN-1346	JAACEH01	JAACEH01	JAACEH01 sp011682595
**GCA_012514125.1**	88.5%	2.4%	87.1%	50.1%	2.98 Mb	JAAYCJ01	JAAYCJ01	JAAZAW01	JAAZAW01 sp012514125
**GCA_012517095.1**	94.3%	3.4%	90.6%	55.3%	4.26 Mb	Sedimentisphaerales	Anaerohalophaeraceae	FEN-1350	FEN-1350 sp012517095
**GCA_012719955.1**	96.6%	1.7%	84.3%	63.5%	5.29 Mb	UBA1845	PWPN01	JAAXZI01	JAAXZI01 sp012719955
**GCA_012729015.1**	96.6%	1.1%	87.1%	66.6%	4.45 Mb	FEN-1346	JAACEH01	JAAYDY01	JAAYDY01 sp012729015
**GCA_012729395.1**	96.6%	2.3%	85.2%	63.4%	5.74 Mb	UBA1845	PWPN01	JAAYDF01	JAAYDF01 sp012729395
**GCA_012729595.1**	96.0%	0.0%	85.5%	57.4%	4.12 Mb	UBA1845	PWPN01	JAAYCU01	JAAYCU01 sp012729595
**GCA_012729675.1**	96.6%	0.0%	87.3%	54.9%	2.80 Mb	Sedimentisphaerales	Anaerohalophaeraceae	T3Sed10-213	T3Sed10-213 sp012729675
**GCA_012729725.1**	97.7%	3.4%	90.9%	59.6%	3.22 Mb	SM23-33	SM23-33	JAAYCO01	JAAYCO01 sp012729725
**GCA_012729815.1**	97.6%	3.2%	90.9%	64.0%	5.47 Mb	JAAYCJ01	JAAYCJ01	JAAYCJ01	JAAYCJ01 sp012729815
**GCA_012744475.1**	100.0%	2.3%	88.4%	62.6%	5.75 Mb	Sedimentisphaerales	SG8-4	PLanc-01	PLanc-01 sp012744475

Table S1. Continued.

**GCA_012965315.1**	94.4%	2.8%	94.5%	43.2%	1.91 Mb	Phycisphaerales	SM1A02	GCA-002718515	GCA-002718515 sp012965315
**GCA_013003065.1**	96.4%	1.3%	90.2%	68.7%	4.57 Mb	Phycisphaerales	SM1A02	JABDLI01	JABDLI01 sp013003065
**GCA_013044065.1**	73.5%	0.0%	87.7%	56.7%	2.80 Mb	Tepidisphaerales	WQYP01	JABEVA01	JABEVA01 sp013044065
**GCA_013044165.1**	96.3%	0.0%	86.6%	56.2%	3.58 Mb	Tepidisphaerales	WQYP01	JABEVB01	JABEVB01 sp013044165
**GCA_013140575.1**	94.9%	2.3%	85.1%	63.4%	5.05 Mb	UBA1845	UBA1845	JABFSN01	JABFSN01 sp013140575
**GCA_013151185.1**	93.5%	0.0%	87.2%	56.6%	3.71 Mb	UBA1845	UBA1845	UBA1845	UBA1845 sp013151185
**GCA_013349435.1**	90.9%	3.4%	86.9%	48.4%	3.14 Mb	Sedimentisphaerales	Anaerohalophaeraceae	QNBT01	QNBT01 sp013349435
**GCA_013349615.1**	89.4%	1.2%	87.7%	49.0%	3.77 Mb	Sedimentisphaerales	SG8-4	CS2-K091	CS2-K091 sp013349615
**GCA_013360675.1**	96.6%	0.0%	91.2%	67.3%	2.92 Mb	Phycisphaerales	SM1A02	JABWBB01	JABWBB01 sp013360675
**GCA_013360695.1**	92.1%	5.1%	88.7%	66.5%	3.84 Mb	Phycisphaerales	SM1A02	JABWBC01	JABWBC01 sp013360695
**GCA_013822085.1**	89.4%	0.0%	89.1%	73.1%	2.26 Mb	Phycisphaerales	SM1A02	WM-009	WM-009 sp013822085
**GCA_014237965.1**	85.4%	0.6%	90.9%	63.3%	3.03 Mb	Phycisphaerales	SM1A02	UBA12014	UBA12014 sp014237965
**GCA_014238125.1**	96.6%	4.7%	94.2%	44.0%	2.07 Mb	Phycisphaerales	SM1A02	GCA-002718515	GCA-002718515 sp014238125
**GCA_014238205.1**	96.6%	0.0%	93.8%	58.0%	2.44 Mb	Phycisphaerales	SM1A02	SP202	SP202 sp014238205
**GCA_014238785.1**	96.0%	0.6%	93.9%	45.1%	1.81 Mb	Phycisphaerales	SM1A02	GCA-002718515	GCA-002718515 sp014238785
**GCA_014238965.1**	95.5%	1.7%	93.8%	56.9%	2.60 Mb	Phycisphaerales	SM1A02	JABAAC01	JABAAC01 sp014238965
**GCA_014379105.1**	76.1%	0.0%	89.5%	59.7%	4.0 Mb	Tepidisphaerales	Tepidisphaeraceae	Tepidisphaera	Tepidisphaera mucosa
**GCA_014379385.1**	87.6%	4.0%	88.1%	66.3%	3.12 Mb	Phycisphaerales	SM1A02	JACMLW01	JACMLW01 sp014379385
**GCA_014381305.1**	93.2%	1.7%	93.5%	45.4%	1.88 Mb	Phycisphaerales	SM1A02	GCA-002718515	GCA-002718515 sp014381305
**GCA_014381385.1**	93.8%	4.0%	94.3%	45.7%	1.89 Mb	Phycisphaerales	SM1A02	GCA-002718515	GCA-002718515 sp014381385
**GCA_014381555.1**	96.6%	1.7%	94.2%	43.8%	1.99 Mb	Phycisphaerales	SM1A02	GCA-002718515	GCA-002718515 sp014381555
**GCA_014384025.1**	94.3%	4.6%	87.1%	46.3%	6.69 Mb	Sedimentisphaerales	SG8-4	NIOZ-UU13	NIOZ-UU13 sp014384025
**GCA_014584315.1**	96.6%	0.0%	91.0%	70.2%	3.33 Mb	Phycisphaerales	SM1A02	CAADGN01	CAADGN01 sp014584315
**GCA_900696425.1**	97.7%	0.0%	89.4%	67.3%	3.62 Mb	Phycisphaerales	SM1A02	CAADGA01	CAADGA01 sp900696425
**GCA_900696465.1**	98.9%	0.0%	88.8%	61.6%	4.14 Mb	UBA1845	UTPLA1	UTPLA1	UTPLA1 sp900696465
**GCA_900696525.1**	90.9%	0.0%	89.4%	69.8%	3.64 Mb	UBA1845	Fen-1342	CAADGL01	CAADGL01 sp900696525
**GCA_900696535.1**	95.5%	1.1%	88.3%	69.0%	4.24 Mb	UBA1845	Fen-1342	CAADGM01	CAADGM01 sp900696535
**GCA_900696545.1**	96.5%	0.0%	90.9%	68.8%	3.09 Mb	Phycisphaerales	SM1A02	CAADGN01	CAADGN01 sp900696545

Table S1. Continued.

**GCA_900696685.1**	93.8%	8.0%	89.5%	65.8%	4.32 Mb	Phycisphaerales	SM1A02	UBA5793	UBA5793 sp900696685
**GCA_903831295.1**	93.8%	2.3%	92.8%	53.2%	2.48 Mb	Phycisphaerales	SM1A02	SYAC01	SYAC01 sp903831295
**GCA_903834785.1**	97.7%	3.5%	86.9%	65.9%	4.32 Mb	Phycisphaerales	SM1A02	CAILKP01	CAILKP01 sp903834785
**GCA_903845195.1**	95.5%	3.4%	87.0%	59.2%	4.62 Mb	Tepidisphaerales	WQYP01	CAIMWO01	CAIMWO01 sp903845195
**GCA_903847315.1**	98.9%	2.3%	90.4%	44.7%	2.44 Mb	Sedimentisphaerales	UBA12454	UBA12454	UBA12454 sp903847315
**GCA_903857265.1**	98.9%	1.2%	89.2%	49.2%	2.81 Mb	Sedimentisphaerales	SG8-4	Fen-1362	Fen-1362 sp903857265
**GCA_903861235.1**	71.4%	1.5%	94.7%	69.6%	2.12 Mb	Phycisphaerales	SM1A02	SYAC01	SYAC01 sp903861235
**GCA_903871935.1**	97.7%	2.0%	89.1%	60.4%	6.32 Mb	Tepidisphaerales	Tepidisphaeraceae	CAIQIQ01	CAIQIQ01 sp903871935
**GCA_903891095.1**	97.7%	0.6%	90.9%	68.0%	3.60 Mb	Phycisphaerales	SM1A02	QWPT01	QWPT01 sp903891095
**GCA_903892515.1**	96.6%	2.3%	85.4%	63.1%	4.89 Mb	SM23-33	SM23-33	CAITIS01	CAITIS01 sp903892515
**GCA_903905665.1**	96.6%	3.4%	88.0%	54.3%	3.16 Mb	UBA1845	UBA1845	CAIVHJ01	CAIVHJ01 sp903905665
**GCA_903911205.1**	96.4%	2.4%	90.8%	61.8%	3.87 Mb	Phycisphaerales	SM1A02	QWPT01	QWPT01 sp903911205
**GCA_903914415.1**	98.9%	3.4%	87.0%	67.5%	4.74 Mb	UBA1845	Fen-1342	Fen-1342	Fen-1342 sp903914415
**GCA_903919715.1**	96.2%	0.0%	94.2%	53.9%	2.90 Mb	Phycisphaerales	SM1A02	F1-60-MAGs104	F1-60-MAGs104 sp903919715
**GCA_903927725.1**	91.5%	3.4%	93.6%	67.7%	2.51 Mb	Phycisphaerales	SM1A02	SYIB01	SYIB01 sp903927725
**GCA_903927855.1**	89.0%	3.5%	89.3%	47.6%	2.05 Mb	Sedimentisphaerales	SG8-4	CAIYOL01	CAIYOL01 sp903927855
**GCA_903929885.1**	97.7%	0.0%	90.2%	68.4%	3.56 Mb	Phycisphaerales	SM1A02	UBA2402	UBA2402 sp903929885
**GCA_903961015.1**	93.9%	1.1%	93.8%	51.4%	2.88 Mb	Phycisphaerales	SM1A02	F1-60-MAGs104	F1-60-MAGs104 sp903961015
**TNR_A1***	83.8%	2.8%	88.1%	66.6%	4.73 Mb	Phycisphaerales	SM1A02	UBA5793	TNR_A1
**TNR_A2***	84.7%	2.3%	88.7%	62.9%	4.01 Mb	UBA1845	UTPLA1	PLA3	TNR_A2
**TNR_N1***	82.1%	1.0%	91.4%	67.4%	3.61 Mb	Phycisphaerales	SM1A02	SYAC01	TNR_N1
**TNR_N2***	80.0%	0.6%	90.3%	70.3%	3.51 Mb	Phycisphaerales	SM1A02	NA	TNR_N2
**TNR_N3***	85.5%	1.2%	87.7%	62.4%	5.16 Mb	UBA1845	UTPLA1	NA	TNR_N3
**TNR_N4***	92.1%	1.8%	88.5%	60.9%	4.93 Mb	UBA1845	UTPLA1	UTPLA1	TNR_N4

Table S2. dbCAN4 grouping of glycans with similar structural features. This table has been copied from J. Zheng, Q. Ge, Y. Yan, X. Zhang, L. Huang, Y. Yin, dbCAN3: automated carbohydrate-active enzyme and substrate annotation. Nucleic Acids Res. 51, W115–W121 (2023).

Glycan	Description	Organisms	Similar to
**agarose**	D-galactose (G) and 3,6-anhydro-L-galactose (L-AHG), G and L-AHG linked by β-(1−4) glycosidic bonds to form a disaccharide-repeating unit, which is then linked by α-(1−3) linkages	red algae	galactan
**carrageenan**	disaccharide-repeating unit same as agarose but sulphated galactan	red algae	galactan
**porphyran**	G and L-galactose-6-sulfate (L-G6S), G and L-G6S linked by β-1,4 linkage to form a disaccharide-repeating unit of G-1,4-(L-G6S), which is then linked by α-1,3 linkage	red algae	galactan
**ulvan**	sulphated rhamnose-3-sulfate (Rha3S) + another sugar forming disaccharide-repeating unit, β-D-GlcA-1,4-α-L-Rha3S, β-L-IdoA-1,4-α-L-Rha3S, β-D-Xyl-1,4-α-L-Rha3S, β-D-Xyl2S-1,4-α- L Rha3S	green algae	polyRha
**laminarin**	β-1,3-linked-D-glucopyranose backbone with β-1,6-linked-D-glucopyranose as a branched chain	brown algae	beta-glucan
**alginate**	β-1,4-D-mannuronic acid (MnA) and α-1,4- L-guluronic acid (GlcA), disaccharide-repeating unit by a 1,4 linkage and arranged as polyMnA, polyGlcA, or polyMnAGlcA blocks	brown algae	polyGlcA
**fucoidan**	highly sulphated polysaccharide, fucose-containing sulfated polysaccharide, α-1,3 linkages and α-1,4 linkages alternately linked with α- L-fucose backbone, and then mannose, galactose, or glucuronic acid to form the branches of the sulphated L-fucose chain	brown algae	polyfucose
**xylan**	β-1,4-xylose backbone with reducing end tetrasaccharide sequence (rhamnose, GalA, xylose), side chains contain arabinose, GlcA, arabinoxylan, glucuronoxylan, glucuronoarabinoxylan	plants and algae	xylan
**arabinogalactan**	beta-galactan backbone and side chain, side chains also contain arabinan, rhamnose, fucose, GlcA, xylose	plants and algae	galactan, arabinan
**cellulose**	β-1,4-glucose	plants and algae	beta-glucan
**beta-mannan**	β-1,4-mannose and β-1,4-glucose backbone, side chain contains galactose	plants and algae	beta-mannan, galactomannan, glucomannan, galactoglucomannan
**mixed-linkage glucan**	β-1,3-1,4-glucose	plants and algae	beta-glucan
**xyloglucan**	β-1,4-glucose backbone, side chains contain xylose, arabinose, galactose, GalA, fucose	plants and algae	beta-glucan

Table S2. Continued.

**lichenin**	β-1,3-1,4-glucose, also known as lichenan or moss starch	lichen (algae, bacteria, fungi composite)	beta-glucan
**callose**	β-1,3-glucose	plants and algae	beta-glucan
**homogalacturonan**	α-1,4-D-galacturonic acid (GalA)	plants and algae	pectin
**rhamnogalacturonan I**	α-linked GalA and Rha disaccharide-repeating unit backbone, side chains include beta-galactan and arabinan, also GlcA and fucose	plants and algae	pectin, galactan, arabinan
**rhamnogalacturonan II**	α-1,4-D-galacturonic acid (GalA) backbone, side chains include arabinose, Dha, Kdo, Rha, apiose, GalA, galactose, xylose, arabinose, fucose	plants and algae	pectin, galactan, arabinan
**isolichenan**	α-1,3-1,4-glucose, also known as isolichenin, cold-water-soluble α-glucan occurring in certain species of lichens	lichen (algae, bacteria, fungi composite)	alpha-glucan
**starch**	α-1,4-glucose with α-1,6 side chain	plants and algae	alpha-glucan
**alpha-galactan**	α-1,4 linked galactose and N-acetylgalactosamine (GalNAc) of exopolysaccharide galactosaminogalactan (fungal GAG), α-galactooligosaccharides (α-GOSs) from plants, also melibiose and raffinose that contain alpha-galactoside	fungi and plant	alpha-galactan, melibiose, raffinose
**alpha-glucan**	starch, glycogen, and malto-oligosaccharides, dextrin, dextran from bacteria, maltose, pullulan, isolichenan, nigerose, trehalose, kojibiose	starch, glycogen	
**sucrose**	α-1,2 linked glucose and fructose	plants and algae	fructan
**alpha-mannan**	α-1,6- and α-1,2-D-mannose backbone	fungi and algae	galactomannan, arabinomannan
**arabinan**	can be found in hydroxyproline-rich glyoproteins (arabinan in extensin and lectin), arabinoxylan, arabinogalactans, pectins	plants and algae	arabinogalactans, pectins
**chitin**	polymer of β-1,4-N-acetylglucosamine	fungi and insects	chitosan
**fructan**	polymer of β-linked fructose with a terminal glucose	plants, bacteria, fungi, algae	inulin, levan, sucrose
**exo-polysaccharide**	diverse polysaccharides on the cell surface of microbes	bacteria, fungi, archaea	capsular polysaccharide, lipopolysaccharides
**galactosaminogalactan**	exopolysaccharide composed of galactose and N-acetylgalactosamine (GalNAc). It is commonly found in the biofilm and cell wall of various fungal species	fungi	exo-polysaccharide

Table S2. Continued.

**host glycan**	broad class of diverse glycans usually connected with proteins or lipids and contain modified mono-sugars such as amino-sugars, e.g., mucin (contain N-acetylgalactosamine (GalNAc), N-acetylglucosamine (GlcNAc), N-acetylneuraminic acid (NeuAc or sialic acid), galactose (Gal), and fucose (Fuc)), hyaluronan, lactose, LacNAc, mucin, blood group B substances, glycosphingolipid, sialic acid, glycosaminoglycan, mucin-type O-glycans, N-glycans, keratan sulfate, human milk oligosaccharides, human milk polysaccharides, heparan sulfate proteoglycans, sulfated glycosaminoglycans, heparin and heparan sulfate, dermatan sulfate	animals, plants	mucin, O-glycans, N-glycans
**peptidoglycan**	long polymers of disaccharides, each containing an N-acetylmuramic acid (MurNAc) and N-acetylglucosamine (GlcNAc) residue, that are linked by β-1,4 glycosidic bonds	bacteria	peptidoglycan
